# Short-term spinal cord stimulation in treating disorders of consciousness monitored by resting-state fMRI and qEEG: The first case report

**DOI:** 10.3389/fneur.2022.968932

**Published:** 2022-10-25

**Authors:** Yi Yang, Qiheng He, Jianghong He

**Affiliations:** ^1^Department of Neurosurgery, Beijing Tiantan Hospital, Capital Medical University, Beijing, China; ^2^Joint Laboratory, Chinese Institute for Brain Research, Beijing, China; ^3^Center of Stroke, Beijing Institute of Brain Disorders, Beijing, China; ^4^Department of Neurosurgery, China National Clinical Research Center for Neurological Diseases, Beijing, China

**Keywords:** spinal cord stimulation, disorders of consciousness, fMRI, EEG, treatment

## Abstract

Disorders of consciousness (DOC) are one of the most frequent complications in patients after severe brain injury, mainly caused by trauma, stroke, and anoxia. With the development of neuromodulation techniques, novel therapies including deep brain stimulation (DBS) and spinal cord stimulation (SCS) have been employed to treat DOC. Here, we report the case of a DOC patient receiving short-term SCS (st-SCS) treatment and showing improvement monitored by resting-state fMRI (rs-fMRI) and quantitative EEG (qEEG). A 35-year-old male with severe traumatic brain injury remained comatose for 3 months. The patient was evaluated using JFK coma recovery scale—revised (CRS-R) and showed no improvement within 1 month. He received st-SCS surgery 93 days after the injury and the stimulation was applied the day after surgery. He regained communication according to instructions on day 21 after surgery and improved from a vegetative state/unwakefulness syndrome to an emergence from a minimally conscious state. To our knowledge, this report is the first published case of st-SCS in a patient with DOC. These results shed light that st-SCS may be effective in treating certain patients with DOC, which may reduce patients' suffering during treatment and lessen financial burden.

## Introduction

Disorders of consciousness (DOC) are one of the most frequent complications in patients after severe brain injury, mainly caused by trauma, stroke, and anoxia (1). Besides drugs, clinicians have tried some non-invasive and invasive neuromodulation technologies, but few of them were proven to improve patients' consciousness levels (2, 3). Patients with DOC impose a burden on medical resources and raise ethical issues. Therefore, it is necessary to develop effective and economic interventions for DOC.

With the development of neuromodulation techniques, novel therapies including deep brain stimulation (DBS) and spinal cord stimulation (SCS) have been employed to treat DOC. In cervical SCS, electrodes were implanted at the midline of the posterior epidural space of C2-C4 level and delivered electric stimulation to the dorsal column. The SCS technology was classically used for pain management, based on the concept of gate control theory (4). Kanno et al. firstly applied this technique to patients with DOC and showed encouraging results (5). However, the previous studies were based on permanent SCS (39286, Medtronic, USA) techniques, which present certain limitations, such as permanent foreign body implantation, high costs of batteries, and difficulty in maintenance. Based on our previous work, SCS with the percutaneous electrode (3777, Medtronic, USA) may also have a positive effect on the rehabilitation of patients with DOC (6–9). The characteristics of SCS with percutaneous electrodes are non-invasive, low cost, and early intervention making it more widely used. Here, we report the first case of percutaneous short-term SCS (st-SCS) in the treatment of a patient with DOC.

## Case description

### Patients

A 35-year-old male presented with severe traumatic brain injury. On admission, he was evaluated using the JFK coma recovery scale—revised (CRS-R, Table 1). He could open his eyes autonomously with no signs of attention or visual tracking and expressed an auditory panic and abnormal posture from pain stimulation. The CRS-R score was 7 points (1-1-2-1-0-2) and was diagnosed as vegetative state/unwakefulness syndrome (VS/UWS). Within 1 month before admission, no improvement in consciousness was observed.

**Table 1:** Patient's CRS-R.

	**T0**	**T1**	**T2**	**T3**	**T4**	**T5**	**T6**
	**Admission**	**Preoperative**	**Day3**	**Day7**	**Day14**	**Day21**	**Day28**
**Auditory function scale**							
4–Consistent movement to command *							
3–Reproducible movement to command *							
2–Localization to sound							
1–Response to auditory stimulation							
0–None							
**Visual function scale**							
5–Object recognition *							
4–Object localization: reaching *							
3–Visual pursuit *							
2–Fixation *							
1–Response to visual stimulation							
0–None							
**Motor function SCALE**							
6–Functional object use							
5–Automatic motor response *							
4–Object manipulation *							
3–Localization to noxious stimulation *							
2–Flexion withdrawal							
1–Abnormal posturing							
0–None							
**Verbal function scale**							
3 Understandable language *							
2 Vocalization/oral movement							
1 Oral reflex movement							
0–None							
**Communication scale**							
2–Functional: Accurate							
1–Non-functional: intentional							
0–None							
**Arousal scale**							
3–Attention							
2–Eye opening without stimulation							
1–Eye opening with stimulation							
0–Unarousable							
Total score	6	7	8	10	14	17	19

*The scale consists of 6 parts, separately representing auditory, visual, motor, verbal, communication, and arousal functions. The total score at each time point is listed. CRS-R, coma recovery scale-revised.

### Surgical procedures

Relatives gave consent to the st-SCS treatment. On the 93 days after the injury, after general anesthesia, the patient was placed in a prone position, and the T7/8 intervertebral space was positioned under C-arm as the puncture point. The electrode (3777, Medtronic, USA) was placed at the C2 level and fixed (Supplementary Figure S1). The day after the st-SCS operation, electric stimulation was applied to the patient's dorsal column with a voltage of 2.5 V, and a frequency of 70 Hz with 120us wave width. The stimulation was performed in 15-min on/15-min off cycles from 8 AM to 8 PM. The overall stimulation lasted for 21 days and then the electrode was removed.

### Postoperative evaluation

Three physicians, who were not in charge of the st-SCS treatment, individually assessed the consciousness level repeatedly. The average of CRS-R was recorded as the final score. Data preprocessing and calculation were consistent with our previous studies and were shown in Appendix S1. Cervical CT scan and VRT reconstruction after SCS implantation are shown in Supplementary Figure S2. On the third day postoperatively (T2), the consciousness level of the patient was slightly improved, mainly manifesting as auditory localization. Then the patient showed visual localization and pain stimulation localization after 7 days. Compliance movement and visual tracking also appeared repeatedly, and he could grasp objects after 14 days. On day 21, he regained the ability to use motor movement expressing whether he ate or urinated. During the final evaluation on day 28, he could communicate according to instructions and was diagnosed as minimally conscious state (MCS). The specific scores are shown in Table 1.

### Imaging results

**Figure 1:**
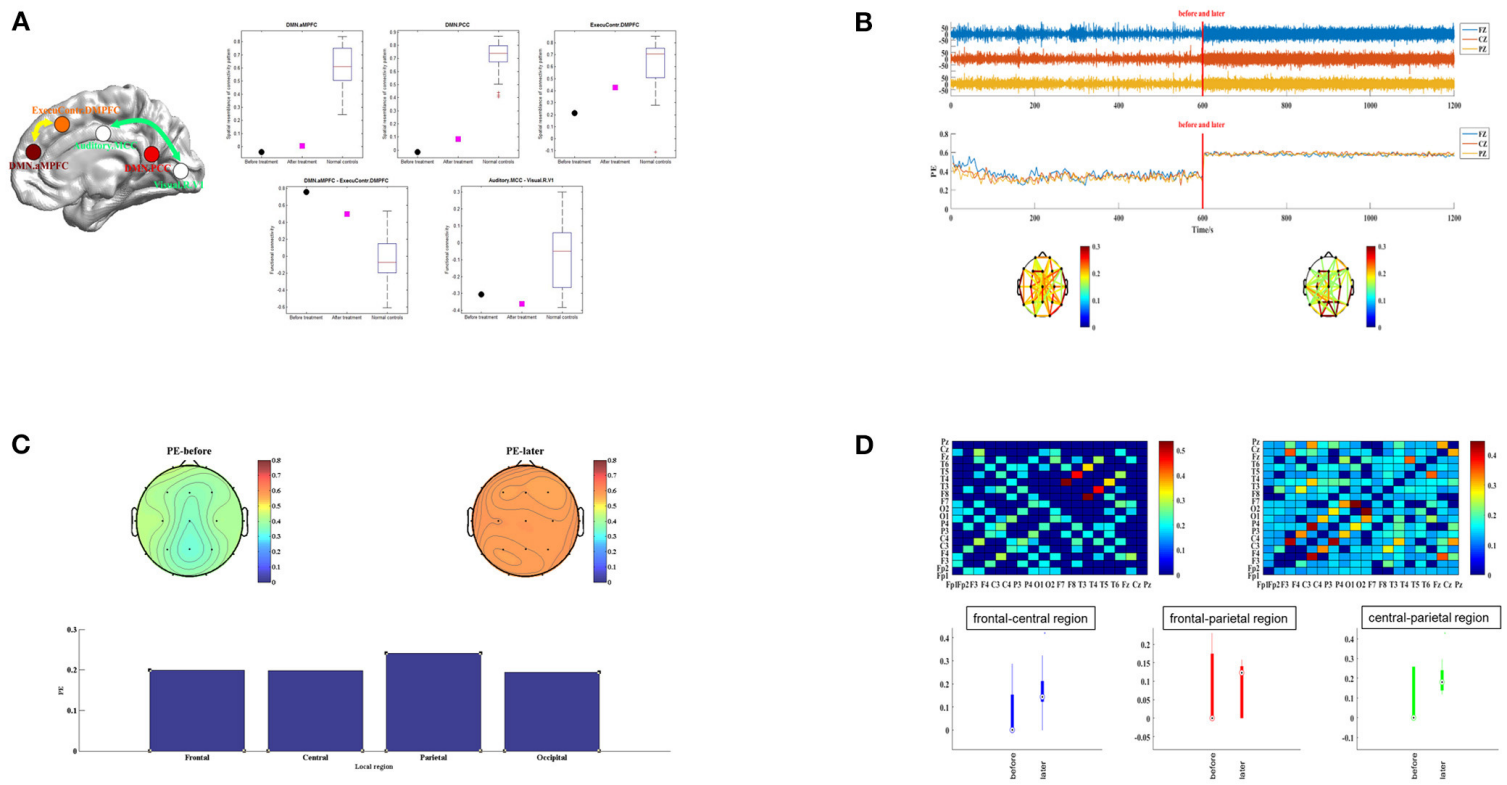
The functional connection mode of the patient before and after treatment. **(A)** The left subplot shows the five imaging features using the package “pDoC.” In the right subplots, the black circles represent the responding imaging measurements of the patient before treatment, and the cyan squares represent the measurements after treatment. The boxplot in each subplot represents the measurement of a group of normal controls. **(B)** EEG activity of patients in different periods. Images of brain amplitude, activity, and complexity before and after st-SCS treatment are presented separately. **(C)** Topographic map distribution and local change rate of PE in different periods. Topographic maps before and after st-SCS treatment are presented in the upper part. PE changes in the frontal, central, parietal and occipital are presented in the below column. **(D)** Recurrence plot of information interaction and brain interval change. The recurrence plot was shown in the upper part. The connectivity changes before and after treatment are compared between the frontal-central, frontal-parietal, and central-parietal brain regions below.

To test the recovery process in the brain function, we performed fMRI and qEEG examinations before and after the st-SCS treatment. The level of preoperative fMRI or qEEG results were considered the baseline level. The preoperative upper limb sensory evoked potential and auditory brainstem response were shown in Supplementary Figure S3. After st-SCS treatment, the patient underwent fMRI and qEEG examinations, and the specific methods are shown in Appendix S1. We found brain area features of the anterior medial pre-frontal cortex (aMPFC) and posterior cingulated cortex (PCC) in the default mode network (DMN), and the dorsal medial prefrontal cortex (DMPFC) in the executive control network (ECN) represented a trend toward the functional connection pattern of normal controls. Functional connectivity between aMPFC in the DMN and DMPFC in the ECN was also changed significantly (Figure 1A). We also found brain activity, amplitude, and rhythm in EEG increased (Figure 1B), and the whole brain ordering entropy changes of patients were compared by the topographic map (Figure 1C). The results showed that the whole brain permutation entropy (PE) increased after treatment, and the change rates of frontal, central, and occipital brain areas were close, while the parietal brain area changed the most significantly. Finally, we found the channel information interaction increased after treatment (Figure 1D). The results indicate that the effect of the new surgical approach of st-SCS in this patient is similar to that of traditional long-term SCS implantation.

## Discussion

Previous literature suggested DOC are the loss of function in certain eloquent brain areas, and the remaining brain functional areas lack sufficient connection or integration to support arousal or awareness. It was generally believed that direct stimulation to the dorsal column could improve such conditions, in ways such as enriching functional communication, motor performance, food intake, and object naming (10). The mechanism of SCS in the treatment of DOC has not yet been fully elucidated, and some studies suggested that the increase of cerebral blood flow and the changes in the expression of neurotransmitters played important roles (11–13). The positive results of fMRI and quantitative EEG indicate that the short-term effect of percutaneous puncture electrodes can activate the key regions and connections of the brain network. Our results indirectly elucidate the mechanism of SCS in treating DOC. The improvement suggests a significant therapeutic effect in an early stage such as st-SCS, which differ from the costly, invasive, and permanent electrode implantation. The surgical approach of temporary electrodes requires no cervical vertebra biting, and the skin incision position is relatively low at T7/8 intervertebral space, reducing the risk and invasiveness of the surgery. Therefore, the st-SCS can be activated the day after implantation while SCS permanent electrode implantation requires a 7–21-day surgical recovery period. Meanwhile, the morphology of the temporary electrode is much simpler than the permanent electrode, so the position of electrode pads can be closer to the midline and less prone to deviation, which is a direct factor affecting the effect of electrical stimulation. The outcome of this case suggests the positive therapeutic effect of st-SCS and short-term electric nerve stimulation for patients with DOC. In this way, the maximal clinical benefit can be obtained while avoiding those resulting side effects.

In this study, the possibility of spontaneous recovery could not be completely ruled out as there was only a single patient and no controls. However, according to previous studies, patients can achieve a better outcome as SCS performed earlier during the course of DOC. Meanwhile, there was no progressive increase or worsening of consciousness during the 4 weeks before the surgery, and a sudden spontaneous recovery after the surgery is considered less likely. Therefore, we considered that it is likely due to st-SCS given the possibility of natural evolution and improvement in this single patient study.

To our knowledge, this is the first case of st-SCS in a patient with DOC. The current case study sheds light on that st-SCS may potential be an effective way of treatment for certain patients with DOC, which may reduce patients' suffering during treatment and release the financial burden. Large-scaled randomized controlled trials are needed to confirm the preliminary findings.

## Data availability statement

The raw data supporting the conclusions of this article will be made available by the authors, without undue reservation.

## Ethics statement

The studies involving human participants were reviewed and approved by Ethics Committee of Beijing Tiantan Hospital, Capital Medical University. The patients/participants provided their written informed consent to participate in this study.

## Author contributions

YY designed the study. QH wrote the manuscript. JH supervised the study. All authors contributed to the article and approved the submitted version.

## Funding

This paper was supported by the National Natural Science Foundation of China (No. 81600919), Beijing Municipal Science and Technology Commission (Nos. Z161100000516165 and Z171100001017162), and Beijing Nova Program (Z181100006218050).

## Conflict of interest

The authors declare that the research was conducted in the absence of any commercial or financial relationships that could be construed as a potential conflict of interest.

## Publisher's note

All claims expressed in this article are solely those of the authors and do not necessarily represent those of their affiliated organizations, or those of the publisher, the editors and the reviewers. Any product that may be evaluated in this article, or claim that may be made by its manufacturer, is not guaranteed or endorsed by the publisher.
