# Supplementary material for: Short-term spinal cord stimulation in treating disorders of consciousness monitored by resting-state fMRI and qEEG: The first case report
Source: Front Neurol. 2022 Oct 25;13:968932. doi: 10.3389/fneur.2022.968932 (PMC9640422; doi:10.3389/fneur.2022.968932)

Appendix S1. Data preprocessing and calculation

The patient was scanned with resting-state fMRI and T1-weighted 3D high-resolution imaging. During the MRI scanning, the patient did not take any sedative or anesthetic drugs. The resting state fMRI acquisition parameters included TR/TE = 2000/30 ms, flip angle = 90˚, axial 32 slices, slice thickness = 3 mm, gap=0.45mm, FOV = 240×240 mm, matrix = 64×64, and 210 volumes.

Preprocessing and connectivity calculation were performed using the tool package “pDoC” (https://github.com/realmsong504/pDOC). The package “pDoC” includes prognostic models based on clinical characteristics and resting state fMRI. Further, using machine learning, the “pDoC” selected five significant imaging features for predicting the DoC patients’s prognosis. Specifically, the brain area features include the anterior medial prefrontal cortex (DMN.aMPFC) and posterior cingulate cortex/precuneus (DMN.PCC) in the default mode network, and the dorsal medial prefrontal cortex in the executive control network (ExecuContr.DMPFC); while the functional connectivity features include the connectivity between the DMN.aMPFC and the ExecuContr.DMPFC, and the one between the middle cingulate cortex in the auditory network (Auditory.MCC) and the right lateral primary visual cortex in the visual network (Visual.R.V1).

Meanwhile, a 30-min EEG was acquired continuously from the patient on 16 channels (BrainAmp 64 MR plus, Brain Products) with the positions of the international 10–20 system. The equipment used sintered Ag/AgCl-pin electrodes. We set a bandpass filtered at DC to 1000 Hz in the recorder, and the EEG signal was digitized at a sampling rate of 2.5 kHz. During the recording, skin/electrode impedance was maintained below 5 kHz. The off-line analysis was performed with EEGLAB 12.0.2.5b, running in a MATLAB environment (Version 2013b, MathWorks Inc., Natick, MA, USA). The EEG data were down-sampled to 500 Hz and bandpass filtered (1–45 Hz). Then, the EEG data were divided into epochs of 10 seconds.

Fig S1. Patient position and surgical procedure. Electrodes are implanted by a puncture at the T7 position to the C2-C4 level, and the wound is minimally invasive.


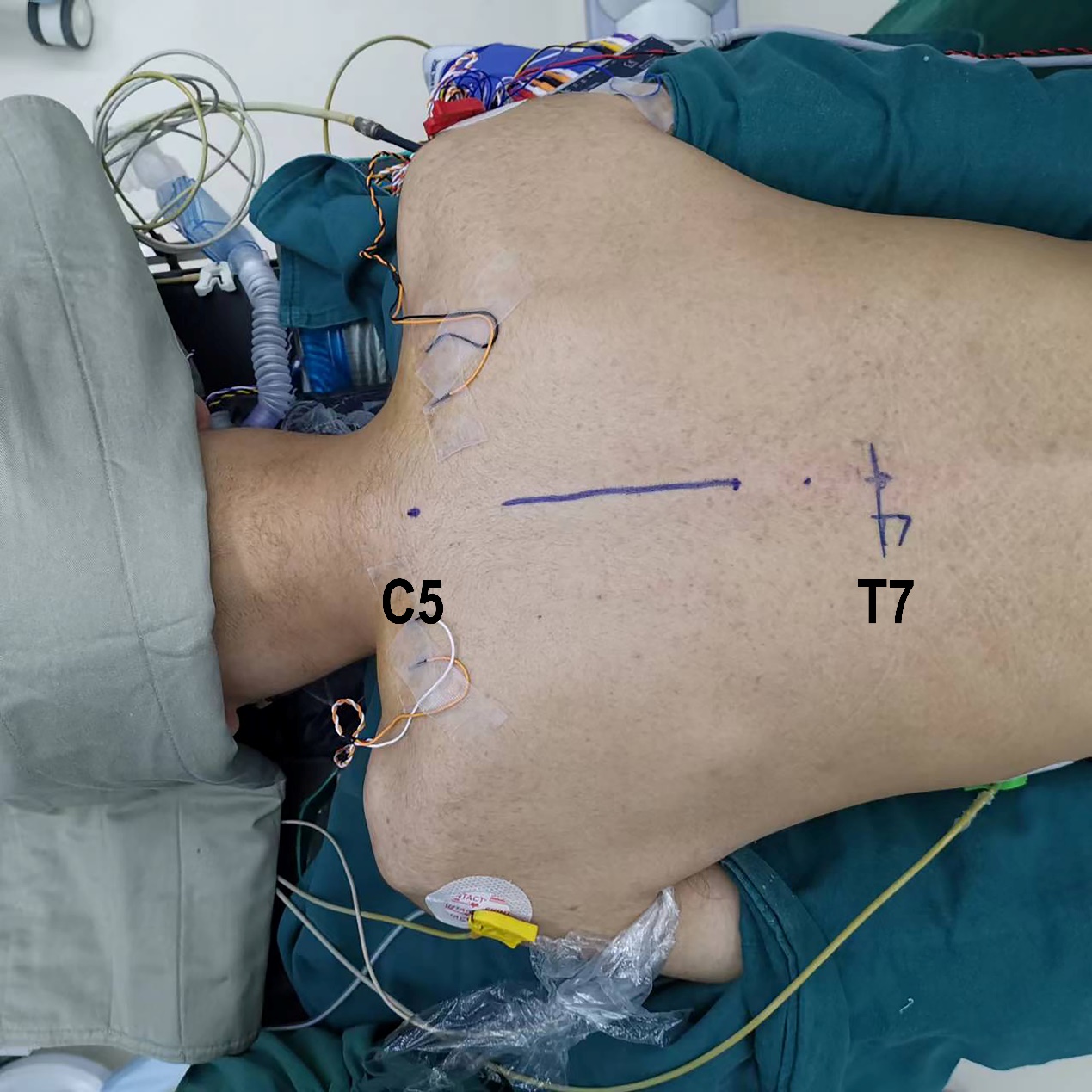


Fig S2. Cervical CT scan after SCS implantation. Image 1 represents the sagittal view of the electrode immediately after implantation. Images 2-4 represent the coronal, sagittal, and axial view of cervical CT, respectively.


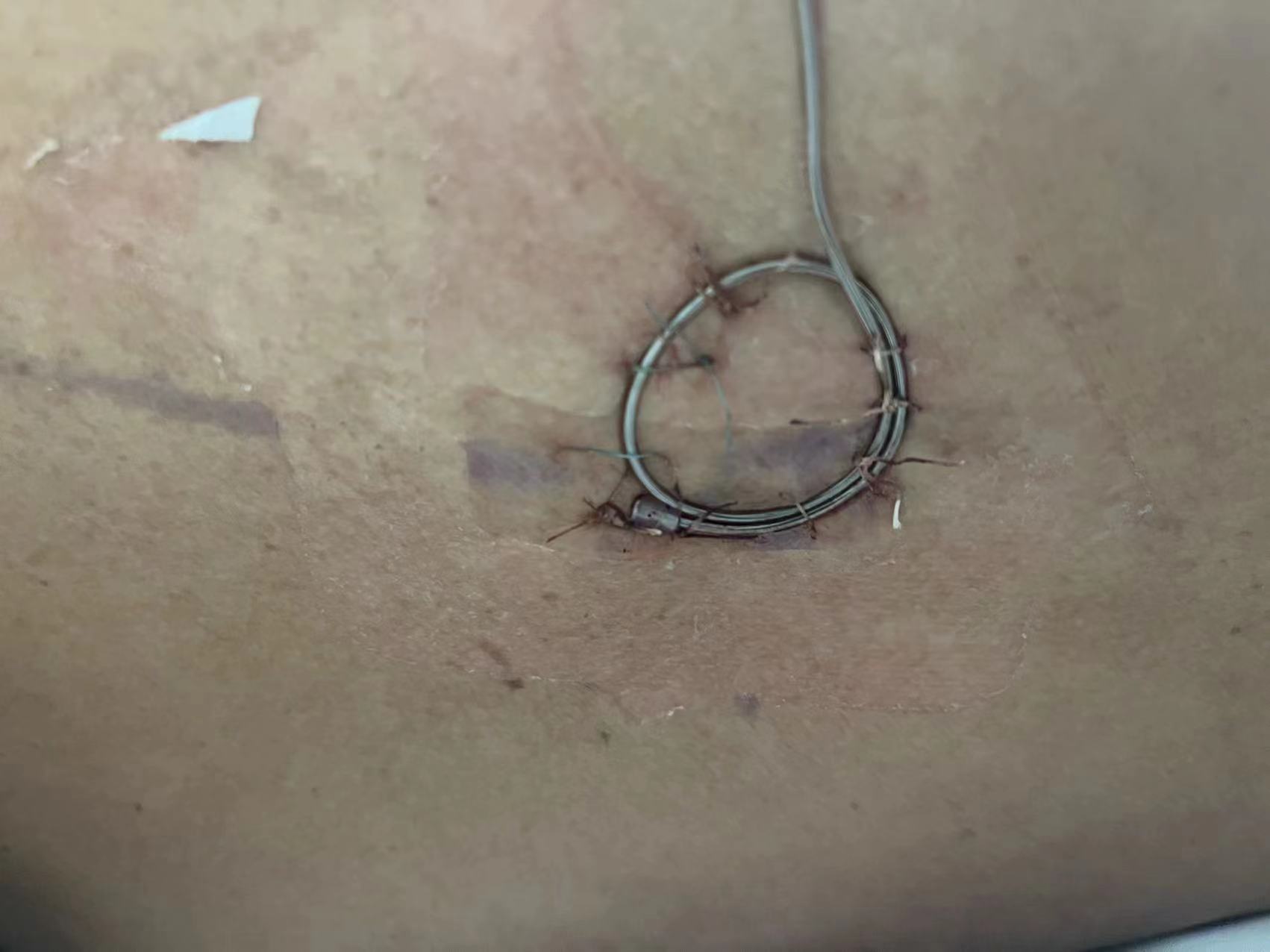


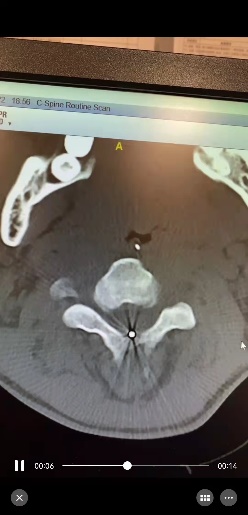

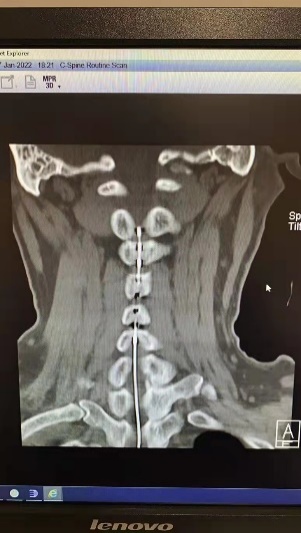

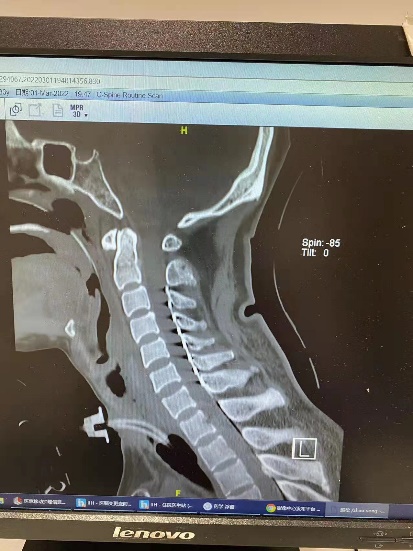

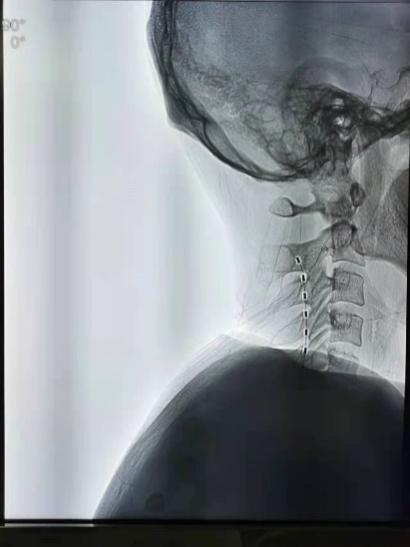


Fig S3. The preoperative upper limb sensory evoked potential and auditory brainstem response. In sensory evoked potential, the latency of the N20 wave in the right cortex was prolonged, and the amplitude of the wave in the right cortex was decreased. In auditory brainstem response, the left V wave, right I wave amplitude decreased, and left V/I < 1.


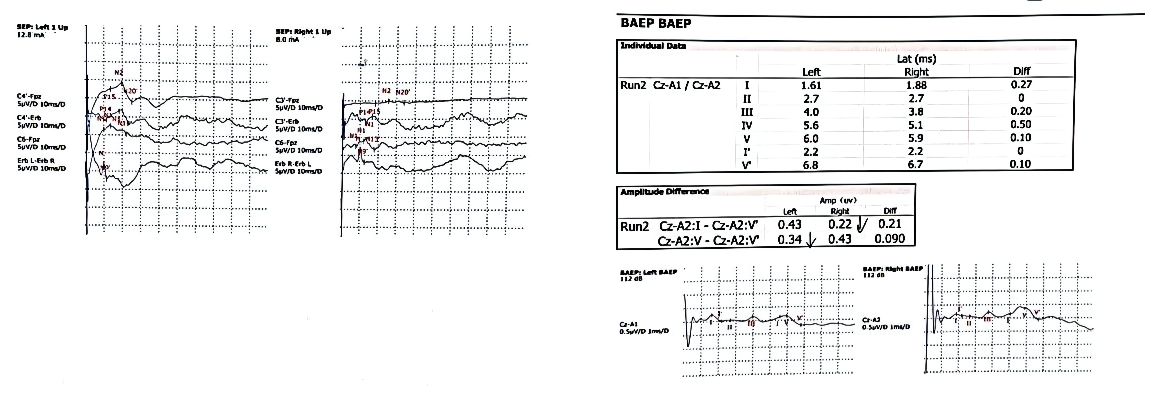

Supplement: Supplementary file 1 [file Data_Sheet_1.docx]
